# Fascin, an actin-bundling protein associated with cell motility, is upregulated in hormone receptor negative breastancer

**DOI:** 10.1054/bjoc.2000.1395

**Published:** 2000-09-04

**Authors:** A Grothey, R Hashizume, A A Sahin, P D McCrea

**Affiliations:** Program in Genes & Development, Department of Biochemistry and Molecular Biology, Department of Pathology; University of Texas, MD Anderson Cancer Center, 1515 Holcombe Blvd, Houston, TX 77030, USA

**Keywords:** fascin, breast cancer, motility, hormone receptor oestrogen, progesterone

## Abstract

Loss of hormone receptor (HR) status in breast carcinomas is associated with increased tumour cell motility and invasiveness. In an immunohistological study of 58 primary breast cancers, oestrogen (ER) and progesterone (PR) receptor levels were inversely correlated with the expression of fascin, an actin-bundling protein associated with cell motility (*P*< 0.0001 and *P* = 0.0019, respectively). In addition, fascin was preferentially expressed in non-diploid tumours (*P* = 0.03). In summary, the upregulation of fascin in HR-negative breast cancers may contribute to their more aggressive behaviour. © 2000 Cancer Research Campaign


*British Journal of Cancer* (2000) **83**(7), 870–873
© 2000 Cancer Research Campaign
doi: 10.1054/ bjoc.2000.1395, available online at http://www.idealibrary.com on IDEAL®

Short Communication

### Fascin, an actin-bundling protein associated with cell motility, is upregulated in hormone receptor negative breast cancer

**A Grothey[1], R Hashizume[1], AA Sahin[2] and PD McCrea[1]**

[1]Program in Genes & Development, Department of Biochemistry and Molecular Biology, [2]Department of Pathology; University of Texas, MD Anderson Cancer Center, 1515 Holcombe Blvd, Houston, TX 77030, USA

**Summary** Loss of hormone receptor (HR) status in breast carcinomas is associated with increased tumour cell motility and invasiveness. In an immunohistological study of 58 primary breast cancers, oestrogen (ER) and progesterone (PR) receptor levels were inversely correlated with the expression of fascin, an actin-bundling protein associated with cell motility ($P < 0.0001$ and $P = 0.0019$, respectively). In addition, fascin was preferentially expressed in non-diploid tumours ($P = 0.03$). In summary, the upregulation of fascin in HR-negative breast cancers may contribute to their more aggressive behaviour. © 2000 Cancer Research Campaign

**Keywords:** fascin; breast cancer; motility; hormone receptor oestrogen; progesterone

The presence of oestrogen and progesterone receptors (ER and PR) is an important prognostic and predictive factor in human breast cancer. Patients with tumours that express ER and PR display a less aggressive phenotype with longer disease-free and overall survival than patients with tumours with no or minimal ER/PR expression (Knight et al, 1977; Early Breast Cancer Trialists' Collaborative Group, 1992). In addition, tumours bearing hormone receptors (HRs) are more likely to respond to hormonal therapy (Jordan et al, 1988). In vitro experiments demonstrated that HR-negative breast cancer cell lines show increased motility and invasiveness and that invasion and metastasis of ER-positive cells can be blocked by ER-antagonists such as tamoxifen (Kantor and Zetter, 1996; Rochefort et al, 1998).

Significantly, the precise molecular mechanisms responsible for the association of increased motility and invasiveness of HR-negative breast cancer cells in vitro, and the more aggressive phenotypes observed in the clinic, are unknown. In this regard, however, increased attention has recently been directed in various cell systems towards proteins having the capacity to modulate actin cytoskeleton dynamics (Keely et al, 1997; Carmeci et al, 1998; Honda et al, 1998).

In this study we analysed the expression of fascin, an actin-bundling protein associated with cell motility, in immunohistochemical sections of breast cancer tissue derived from HR-negative and HR-positive tumours. Our results demonstrate that fascin is significantly upregulated in ER- and PR-negative breast cancer, conceivably contributing to the more malignant phenotype of HR-negative tumours via effects upon actin based structures required in cell motility and/or invasion processes.

*Received 18 June 1999*
*Revised 5 April 2000*
*Accepted 8 May 2000*

*Correspondence to:* Pierre D McCrea

### MATERIAL AND METHODS

Tissue specimens were obtained from 58 female patients (14 pre-, 44 postmenopausal, median age 61 yrs, range 33–85 yrs) with primary invasive breast cancer. None of the patients had received preoperative irradiation or chemotherapy. The 58 tumours were histologically categorized as 38 ductal, 7 lobular, 7 medullar, 2 mucinous, and 4 tubular carcinomas according to the World Health Organization classification. Normal, non-malignant breast tissue taken from sites adjacent to cancerous lesions were also collected for immunostaining. Patient and tumour characteristics are listed in Table 1.

Formalin-fixed, paraffin-embedded specimens were cut, dewaxed and blocked with 1% (w/v) bovine serum albumin for 30 minutes. Sections were then incubated with a mouse monoclonal anti-human fascin antibody (1:20 dilution; Dako Corp, Carpinteria, CA) for 16 h at 4°C and immunostained using the alkaline phosphatase anti-alkaline phosphatase (APAAP) immune complex method (Universal APAAP kit, Dako) (Pinkus et al, 1997). The binding products were visualized with alkaline phosphatase substrate containing naphthol AS-MX phosphate, Fast Red TR reagents and levamisole as chromogen. Negative controls were carried out by replacing the primary antibody with normal mouse lgG1. Slides were counterstained with haematoxylin and mounted with glycergel (Dako).

Immunohistochemical staining was independently scored by two observers without the knowledge of all other clinicopathologic features. Discrepant cases were reviewed to achieve a consensus. The extent and pattern of the staining were each evaluated. Cases were scored as positive when more than 5% of the tumour cells showed positive staining. Presence of immunoreactivity in more than 50% of cells was scored as diffuse positive. Borderline staining was defined as positive staining confined to the outer edges of the tumour clusters. Non-borderline staining was defined as presence of diffuse staining without apparent spatial localization.

**Table 1** Patient and tumour characteristics

| | Number of patients | |
|---|---|---|
| Total | 58 | |
| Median age (range) | 61 yrs | (33–85) |
| Premenopausal | 14 | |
| Postmenopausal | 44 | |
| Histology (fascin positive tumours) | | |
| ductal | 38 | (8) |
| lobular | 7 | (1) |
| medullary | 7 | (2) |
| mucinous | 2 | (0) |
| tubular | 4 | (1) |
| Stage | | |
| T1 | 22 | |
| T2 | 27 | |
| T3 | 2 | |
| T4 | 7 | |
| N0 | 29 | |
| N1 | 21 | |
| N2 | 7 | |
| NX | 1 | |
| M0 | 51 | |
| M1 | 7 | |
| Receptor status | | |
| ER positive | 48 | |
| ER negative | 10 | |
| PR positive | 44 | |
| PR negative | 14 | |

Oestrogen and progesterone receptor levels were determined using a routine enzyme immunoassay (Leclercq et al, 1986). Tumours were classified as ER- or PR-positive if receptor levels were ≥10 fmol/mg (Sigurdsson et al, 1990). Ploidy of tumour cells was determined by flow cytometry using a method described elsewhere (Vindelow et al, 1983).

Statistical analysis was carried out using the Chi-Square-Test unless otherwise mentioned. Statistical significance was assumed if $P < 0.05$.

### RESULTS

Forty-eight of 58 breast cancers had ER levels ≥ 10 fmol/mg (= ER-positive), 43 of which (93%) were negative for fascin. In contrast, in 7 of 10 ER-negative tumours (70%) fascin expression could be readily demonstrated via immunohistochemical staining. Statistically, this difference was highly significant ($P$ = 0.0002, Fisher's exact test). An inverse correlation between PR and fascin expression was likewise observed as 39 of 44 (88.6%) PR-positive and only 7 of 14 (50%) PR-negative tumours showed a negative staining reaction for fascin ($P$ = 0.0047, Fisher's exact test) (Figure 1A). All 6 tumours negative for ER and PR were fascin positive in contrast to only 4 of 40 (10%) tumours positive for ER and PR (Figure 1B). As expected, ER and PR levels were well correlated ($P$ = 0.0032, Spearman test). Fascin expression showed no significant correlation with menopause status, tumour stage, histology, grading, number of lymph nodes involved, presence of metastasis at time of surgery, CEA and CA15–3 levels. However, flow cytometry analysis revealed that fascin negative tumors were more likely diploid (26 of 43 (60.5%)) than non-diploid (17 of 43 (39.5%)), a difference that reached statistical significance ($P$ = 0.03). No significant correlation was found between histological tumour grading and ploidy ($P$ = 0.12). Immunohistochemically, fascin positivity appeared as cytoplasmic staining with a marked enhancement in areas of tumour–host interaction in most samples (Figure 2).

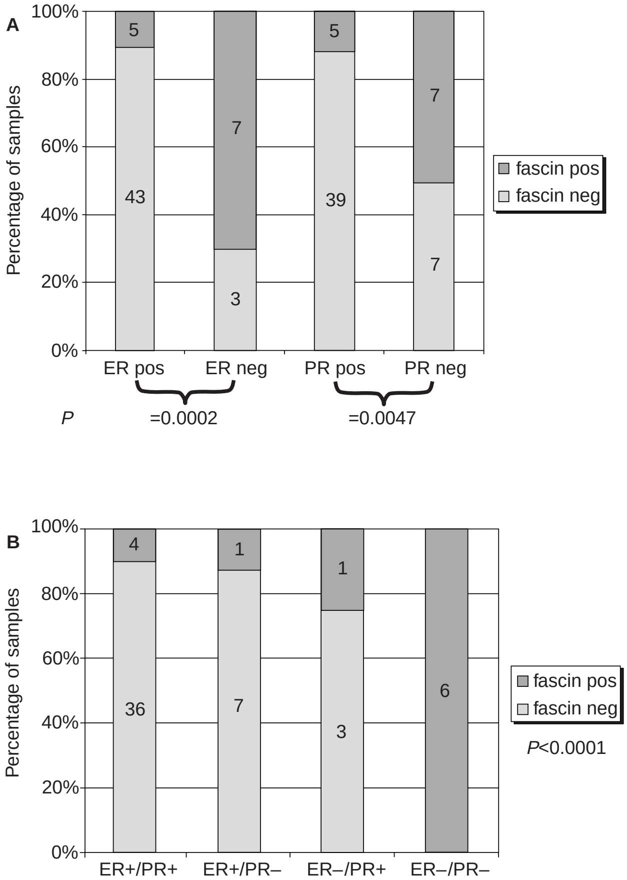

**Figure 1** Differential expression of fascin in ER- or PR-positive and -negative breast cancers. Numbers in bars reflect the absolute number of cases. Statistical evaluation by Fisher's exact test (panel A) or Chi-square test (panel B)

### DISCUSSION

It is well-known that ER-negative breast cancers display a more aggressive behaviour than ER-positive tumours. As regulation of the actin cytoskeleton plays a crucial role in cell motility and cancer invasion, molecular modulators of actin dynamics might be anticipated to contribute to the malignant phenotype of cancer cells. In the context of normal cells, for example, it is well accepted that the plasticity of the cytoskeleton is modulated via the activities of actin-associated proteins (Mitchison and Cramer, 1996).

Proteins that are capable of bundling or binding actin filaments are numerous and include fimbrin, various tropomyosin isoforms, gelsolin, α-actinin, and α-catenin (Otto, 1994). Early studies identified changes in the organization of the cytoskeleton and junctional proteins in cancer cells, largely indicating a reduction in the expression of actin associated proteins (Ben-Ze'ev, 1985; Asch et al, 1996). In addition, it has been shown that restoration of vinculin and α-actinin expression in cancer cells results in decreased tumorigenicity and metastatic properties (Rodriguez Fernandez et al, 1992; Gluck et al, 1993).

© 2000 Cancer Research Campaign

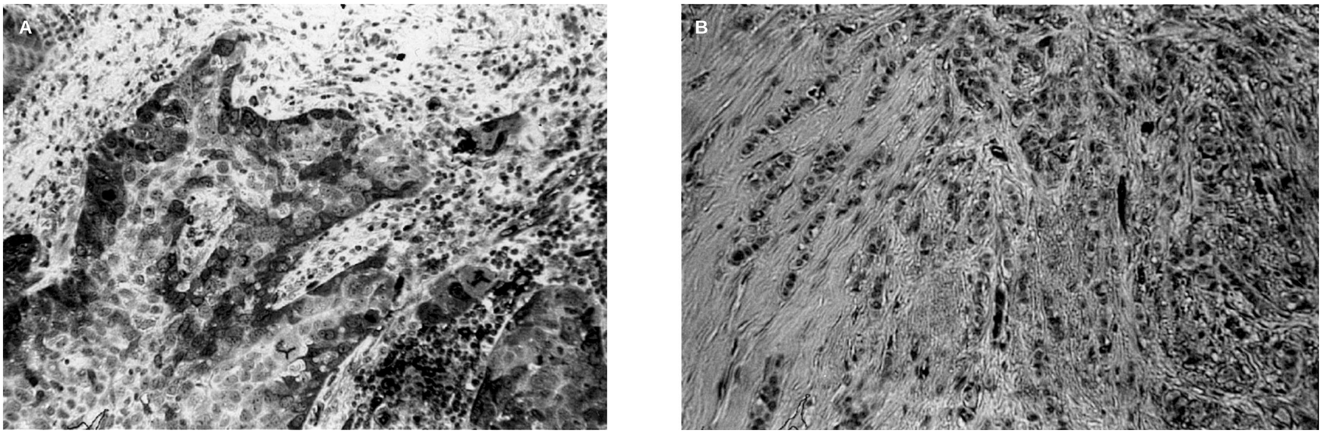

**Figure 2** Immunohistological expression of fascin in two different invasive ductal carcinomas (APAAP method, 40×). Note the cytoplasmic staining and enhanced reaction at the tumour–host border in the fascin-positive carcinoma (panel A). A tumour with negative staining for fascin is shown in panel B. Note that endothelia of small vessels are fascin-positive, which validates the immunohistochemistry reaction in fascin-negative tumours

More recently, however, various proteins that modulate dynamic properties of the actin cytoskeleton have been associated with an increased malignant potential in tumour cells, namely the small G-proteins Rac, Rho, and cdc42, the guanine nucleotide exchange factor Tiam-1, EMS1, and actinin-4 (del Peso et al, 1997; Hordijk et al, 1997; Hui et al, 1997; Keely et al, 1997; Honda et al, 1998). Further, the actin-binding protein moesin, a member of the talin-4.1 superfamily was found to be associated with the ER-negative breast cancer phenotype (Carmeci et al, 1998). Similar to fascin, moesin is localized in filopodia and other subcellular structures engaged in active cell motility processes (Amieva and Furthmayr, 1995).

Fascin possess two actin-binding domains within a single molecule, permitting tight packing of filamentous actin (Tilney et al, 1995). Fascin has been found in highly motile and dynamic subcellular structures such as microspikes, lamellipodia, and filopodia (Edwards and Bryan, 1995). The overexpression of native human fascin in a pig epithelial cell line (LLC-PK1) was associated with reduced cell–cell junction integrity, the development of a fibroblastic phenotype, and increased cell motility (Yamashiro et al, 1998), which has recently been correlated with phosphorylation of fascin on serine 39 (Adams et al, 1999). Reduced junctional integrity has likewise been associated with increased fascin expression in the absence of glucocorticoids (Wong et al, 1997), again consistent with reduced adhesion and increased motility in cells expressing relatively greater levels of fascin. Our own experiments, to date published in abstract form, have revealed that fascin exhibits highly increased levels in breast cancer cell lines over-expressing the receptor tyrosine kinase and prognostic indicator c-erbB-2/HER-2, and that such cells exhibit dramatically increased cell dynamics and in vitro motility (Grothey et al, in press).

In this study we demonstrate that the expression of fascin is clearly associated with the absence of ER and PR in invasive breast carcinomas. Moreover, fascin is preferentially expressed in non-diploid tumours although no correlation with the histological grading could be observed. Histologically, fascin staining is often enhanced at the leading edges of infiltrating tumours, which indicates its role as a pathogenic factor for tumour cell invasion. The molecular mechanism leading to the increased expression of fascin in HR-negative breast carcinomas is presently unknown, and work is in progress to analyse the effect of steroid hormones on fascin's transcriptional regulation (GenBank accession number U90355, submitted February 24, 1997, by Tubb BE, Lee R, and Bryan J).

In conclusion, ER-and PR-negative breast cancers are characterized by an increased expression of the actin-bundling, motility-associated protein fascin. It is conceivable that fascin may serve as a downstream cytoskeletal effector contributing to the more aggressive/malignant phenotype of HR-negative breast cancer.

### ACKNOWLEDGEMENTS

Axel Grothey was supported by a grant of the German Cancer Foundation (Mildred-Scheel-Stiftung). We also gratefully acknowledge the current and past sources of support that have contributed to the execution of this work: NIH RO1 Grant GM 52112; Texas ARP Grant 15-135; March of Dimes Basil O'Connor Grant 5-0926; Kleberg Foundation Award; and CCSG Developmental Funds CCSG-CA 16672.

### REFERENCES

Adams JC, Clelland JD, Collett GD, Matsumura F, Yamashiro S and Zhang L (1999) Cell–matrix adhesions differentially regulate fascin phosphorylation. *Mol Biol Cell* **10**: 4177–4190

Amieva MR and Furthmayr H (1995) Subcellular localization of moesin in dynamic filopodia, retraction fibers, and other structures involved in substrate exploration, attachment, and cell–cell contacts. *Exp Cell Res* **219**: 180–196

Asch HL, Head K, Dong Y, Natoli F, Winston JS, Connolly JL and Asch BB (1996) Widespread loss of gelsolin in breast cancers of humans, mice, and rats. *Cancer Res* **56**: 4841–4845

Ben-Ze'ev A (1985) The cytoskeleton in cancer cells. *Biochim Biophys Acta* **780**: 197–212

Carmeci C, Thompson DA, Kuang WW, Lightdale N, Furthmayr H and Weigel RJ (1998) Moesin expression is associated with the estrogen receptor-negative breast cancer phenotype. *Surgery* **124**: 211–217

del Peso L, Hernandez-Alcoceba R, Embade N, Carnero A, Esteve P, Paje C and Lacal JC (1997) Rho proteins induce metastatic properties in vivo. *Oncogene* **15**: 3047–3057

Early Breast Cancer Trialists' Collaborative Group (1992) Systemic treatment of early breast cancer by hormonal, cytotoxic, or immune therapy. 133 randomised trials involving 31 000 recurrences and 24 000 deaths among 75 000 women. Early Breast Cancer Trialists' Collaborative Group. *Lancet* **339**: 1–15

Edwards RA and Bryan J (1995) Fascins, a family of actin bundling proteins. *Cell Motil Cytoskeleton* **32**: 1–9

© 2000 Cancer Research Campaign

Gluck U, Kwiatkowski DJ and Ben-Ze'ev A (1993) Suppression of tumorigenicity in simian virus 40-transformed 3T3 cells transfected with α-actinin cDNA. *Proc Natl Acad Sci USA* **90**: 383–387

Grothey A, Hashizume R, Ji H, Tubb BE, Patrick CW, Yu DH, Mooney EE and McCrea PD. C-erbB-02/HER-2 upregulates fascin, an actin-bundling protein associated with cell motility in human breast cancer cell lines. *Oncogene* (in press)

Honda K, Yamada T, Endo R, Ino Y, Gotoh M, Tsuda H, Yamada Y, Chiba H and Hirohashi S (1998) Actinin-4, a novel actin-bundling protein associated with cell motility and cancer invasion. *J Cell Biol* **140**: 1383–1393

Hordijk PL, ten Klooster JP, van der Kammen RA, Michiels F, Oomen LCJM and Collard JG (1997) Inhibition of inhibition of invasion of epithelial cells by Tiam1-Rac signaling. *Science* **278**: 1464–1466

Hui R, Campbell DH, Lee CS, McCaul K, Horsfall DJ, Musgrove EA, Daly RJ, Seshadri R and Sutherland RL (1997) EMS1 amplification can occur independently of CCND1 or INT-2 amplification at 11q13 and may identify different phenotypes in primary breast cancer. *Oncogene* **15**: 1617–1623

Jordan VC, Wolf MF, Mirecki DM, Whitford DA and Welshons WV (1988) Hormone receptor assays: clinical usefulness in the management of carcinoma of the breast. *Crit Rev Clin Lab Sci* **26**: 97–152

Kantor JD and Zetter BR (1996) Cell motility in breast cancer. *Cancer Treat Res* **83**: 303–323

Keely PJ, Westwick JK, Whitehead IP, Der CJ and Parise LV (1997) Cdc42 and Rac1 induce integrin-mediated cell motility and invasiveness through Pl(3)K. *Nature* **390**: 632–636

Knight WA, Livingston RB, Gregory EJ and McGuire WL (1977) Estrogen receptor as an independent prognostic factor for early recurrence in breast cancer. *Cancer Res* **37**: 4669–4671

Leclercq G, Bojar H, Goussard J, Nicholson RI, Pichon MF, Piffanelli A, Pousette A, Thorpe S and Lonsdorfer M (1986) Abbott monoclonal enzyme immunoassay measurement of estrogen receptors in human breast cancer: a European multicenter study. *Cancer Res* **46**: 4233s–4236s

Mitchison TJ and Cramer LP (1996) Actin-based cell motility and cell locomotion. *Cell* **84**: 371–379

Otto JJ (1994) Actin-bundling proteins. *Curr Opin Cell Biol* **6**: 105–109

Pinkus GS, Pinkus JL, Langhoff E, Matsumura F, Yamashiro S, Mosialos G and Said JW (1997) Fascin, a sensitive new marker for Reed-Sternberg cells of Hodgkin's disease. Evidence for a dendritic or B cell derivation? *Am J Pathol* **150**: 543–562

Rochefort H, Platet N, Hayashido Y, Derocq D, Lucas A, Cunat S and Garcia M (1998) Estrogen receptor mediated inhibition of cancer cell invasion and motility: an overview. *J Steroid Biochem Mol Biol* **65**: 163–168

Rodriguez Fernandez JL, Geiger B, Salomon D, Sabanay I, Zoller M and Ben-Ze'ev A (1992) Suppression of tumorigenicity in transformed cells after transfection with vinculin cDNA. *J Cell Biol* **119**: 427–438

Sigurdsson H, Baldetorp B, Borg A, Dalberg M, Ferno M, Killander D and Olsson H (1990) Indicators of prognosis in node-negative breast cancer. *N Engl J Med* **322**: 1045–1053

Tilney LG, Tilney MS and Guild GM (1995) F actin bundles in Drosophila bristles. I. Two filament cross-links are involved in bundling. *J Cell Biol* **130**: 629–638

Vindelow L, Christensen I and Nissen N (1983) A detergent-trypsin method for the preparation of nuclei for flow cytometric DNA analysis. *Cytometry* **3** 323–327

Wong V, McCrea PD and Firestone GL (1997) Fascin protein down regulation is required for glucocortiocoid-induced adherens and tight junctions formation in mammary epithelial cells. *Mol Biol Cell* **7**: 'Special Poster' Abstract H8

Yamashiro S, Yamakita Y, Ono S and Matsumura F (1998) Fascin, an actin-bundling protein, induces membrane protrusions and increases cell motility of epithelial cells. *Mol Biol Cell* **9**: 993–1006

© 2000 Cancer Research Campaign 

